# Michigan State Dental Association

**Published:** 1872-11

**Authors:** D. C. Hawxhurst


### PROCEEDINGS OF SOCIETIES.

### Michigan State Dental Association.

This association met at Grand Rapids, October 10th, 1871. with Dr. E. S. Holmes in the chair. There not being a quorum present, an informal discussion took place on the subject of extracting teeth, in which the strongest grounds were taken against the operation except as a last resort. Several members maintained that all remaining roots should be rendered healthy and either pivoted or spliced with gold as far as possible.

On the day following, the President, Dr. E. S. Holmes, read an essay, welcoming the members of the association and the profession in general.

New members were admitted as follows: Drs. L. M. Hutchinson, of Big Rapids; J. B. Smith, of Union City; J. H. Wooley, of Coldwater; G. H. Cummings, of South Haven. A motion was carried to reinstate old members who were behind in payment of yearly dues on the payment of a simple initiation fee.

Dr. Watling, Chairman of the Committee on Anatomy, gave a verbal report, showing the necessity of thoroughness in this department. He caused it to appear that a knowledge of the tissues operated upon is the basis of all the treatment; that the first thing for the student to learn is the normal state of these tissues; that he must follow this up by studying their diseased states; that after this he must possess himself of their origin and development; and that a knowledge of the dental tissues alone is insufficient. The student who aspires to be thorough in the mastery of his chosen profession must pass in review the *entire structures* of the human system.

Some lively discussion followed this report, in which it was generally maintained that nothing in the profession is more reprehensible than the ignorance of general and histological anatomy exhibited by some of its members.

Dr. Hutchinson made some remarks concerning a new preparation of phosphate of lime, which had been prepared by

Dr. King, of Kalamazoo, and was on exhibition by Dr. Bannister. He requested Dr. Bannister to read a paper on the subject of phosphate of lime in its application to diseased states of the dental and osseous tissues, which had also been sent in by Dr. King. The paper opened with a record of observations, showing the promptness with which the lime salts will alleviate certain states of irritation in the mucous membrane and by consequence relieve certain kinds of dyspepsia The next point made was, that irritation of the mucous membrane is often propagated to the periosteum of the teeth and to the pulp membrane, and that in this way a disease which is wholly confined to the nutritive system may extend itself to the dental organs and constitute the basis of their final destruction. He now proceeded to show that the phosphate of lime not only allays irritation of the mucous membrane, but that it promptly removes those secondary effects which sometimes prove so troublesome in the alveola-dental membrane. The rest of the paper was devoted to a description of Dr. King's method of preparing phosphate of lime. The lozenges, which he presented to the association, were designed to furnish this substance in a state of extreme division. A considerable portion of pure sugar of milk had been thoroughly triturated with a smaller quantity of phosphate of lime, and the whole made up into lozenges as the most convenient form for administration.

Dr. Davis said that he supposed mineral substances could not be assimulated unless first organized in the vegetable; that Huxley teaches this distinctly, for he has said that all matter must take the form of protoplasm before it can serve as nutrition in the animai organism. He said that in his own view he knew of but a single instance which did not accord with Huxley's position, that of the benefit we derive from the use of mineral waters.

Dr. Hutchinson expressed his faith in Huxley's position, and suggests that the benefit derived from mineral waters is no proof that their mineral ingredients are assimilated.

Dr. Douglas thinks that phosphate of lime may modify states of health through the dynamic power of the drug and without being assimilated; because mineral wateis influence the health we must not conclude that their ingredients are builded into animal tissues as a portion of their constituent parts.

Committee on Pathology: There being no report ready on this subject, Dr. Hawxhurst made some remarks on the nature and origin of pathological action. He believed, with Huxley, Virchow, Beale, and Carpenter, that all vital action originates in the cell; that in order to know much about it we must study it in the cell; that disease, too, which is merely a modified vital action, is first manifested in the cell; that morbid conditions can never be understood without referring them to pathological state in the ultimate cell; and that the dental student, as soon as he has acquainted himself with the more obvious facts relating to animal tissues, should study their microscopic characters; should study the *career* of the cell, its anatomy, physiology and pathology. He maintained that disease must have gone a long way before it can become visible in the tissues to the unassisted eye, and that by consequence the microscope may reveal disease long before it has become a source of discomfort to the patient. He gave some account of Virchow's great contribution to this department of pathology, and urged the necessity of a profound acquaintance with normal vital action as a basis on which to study all departures from the normal standard.

After some discussion on this and kindred topics, Dr. Douglas was asked the question whether he had found "hammamelis virginica" as useful in controlling dental hemorrhage as his former experience detailed to the association had led him to expect? He stated that this drug had in his hands been uniformly efficient in the control of arterial hemorrhage, and that it had more than met his expectation when used for this purpose. He uses the offiicinal tincture applied on a twist of cotton directly to the part. In bad cases he gives it internally in doses from one to sixty drops, and awaits its effect with perfect confidence. Its action is sometimes wonderful. Relies upon plugging as a next resort, and if these measures fail he advances to persulphate of iron.

Dr. Dart plugs the alveola cavity with sponge, and with this simple remedy is uniformly successful in arresting hemorrhage. Thinks the elasticity of the sponge contributes to the tightness of the plug. The discussion having turned upon cleaning teeth as a remedy for sensitive dentine, Dr. Dort called attention to the corroding effect of rancid butter; the buttyric acid attacks dentine and seizes upon its lime salts.

Dr. Douglas concurred in this, and stated that the acid originating in a dyspeptic stomach might often effect decomposition of exposed dentine. This action might prove very destructive if prolonged over a period of several years. In his own case he had found common salt a remedy for this condition, and finds it much more effective than alkalies. Takes a pinch of salt into the mouth after the meal is over and gradually dissolves and swallows it. Commenced this treatment a year ago, and has enjoyed excellent health ever since. The parotid gland always discharges an acid saliva in conjunction with an acid state of the stomach. Finds two drachms of salt, more or less, a perfect remedy.

Dr. J. J. Robbins described a case in which the permanent lower central incisors were erupted at the fortieth month. In answer to a question he stated that he believed that the ex cessive use of mercury as a medicine might produce deleterious effects in the next generation of children. A long discussion followed between Drs. Hutchinson, Douglas, and Robbins, from which it appeared that mercurial vapor applied to an exposed nerve would result in its hasty destruction. From their remarks and the cases brought forward it seemed that the subject is involved in great mystery.

Dr. Cummings asked for some hints concerning the treatment of diseased gums. A lively discussion followed by Drs. Holmes, Dart, Hawxhurst, and Douglas, during which it was argued that drugs are less important in the treatment of gums than cleanliness; that chloride of zinc, iodine, calendula, aconite, carbolic acid, or gum myrrh will each and all prove very efficient in rendering diseased gums healthy, provided only that the necks of the teeth are kept absolutely free from foreign matters; that cleanliness alone will thoroughly and perfectly accomplish the same result in most cases; that the curative influences of drugs often lie in the cleansing treatment preparatory to their exhibition, and finally that all we have to do in most cases of diseased gums is to remove causes of irritation, and leave nature free to restore the lost integrity of the tissue.

Dr. Hawxhurst desired to call out an expression in regard to cases in which the pulp has been long dead in a sealed canal without abscess.

Dr. Douglas cleanses the canal in these cases and fills at once; if soreness results gives "mercurious vivus" and repeats the dose until the inflammation subsides. Has not had a case go on to suppuration during the last five years. Finds those cases in which the abscess has already appeared become chronic, more irritable than those in which the pulp chamber has been long sealed with the remains of a pulp in it. Has a great deal of faith in "mercurious vivus" as a local remedy in these cases.

Dr. Watling asked if Dr. Douglas has ever attempted to produce an alveolar abscess by giving "mercurious vivus." If it would do this administered in large doses he thinks he would believe that it would cure the same given in homeopathic doses.

Dr. Douglas reiterated his faith in the "mercurious vivus" as based on long experience.

Dr. Watling says that ninety cases in a hundred will be cured by a little counter-irritation with saturated solution of iodine in creosote, or a little anti-phlogistic treatment with blue pills or a cathartic. He invited those who did not want to be homeopathists to find a refuge with him in a substantial system of medication which years of practice had proved to be both safe and efficacious.

Dr. Douglas described a new method of extracting the root of a tooth when broken deep into the jaw. He stated that he had sometimes found great difficulty in getting hold of these fragments and effecting their dislodgement. He had recently in important cases cut an annealed steel instrument with a screw plate, to fit the drilled canal of the root. After inserting the instrument the root may be extracted with more certainty and less pain than by any other method.

Dr. Hutchinson described a case in which the alveolar absorption, after the extraction of the entire set of upper teeth, was complete, but the jaw still too prominent in front to admit anything like an artistic adaptation of an artificial denture. He asked the opinion of the association concerning the best method of treatment, and finally asked if it would be admissible in any case to dissect off the now smooth and healthy gum, and cut away enough of the jaw and process to render possible the insertion of well-adapted teeth?

Dr. Watling remarked that few dentists have the requisite boldness to do this; few the necessary anatomical knowledge.

Dr. Stone says that he always removes such portions of superior maxilla and alveolus as he conceives desirable in cases of recent extraction, and before absorption, of course. In these cases he does not think it required much nerve or boldness. The required anatomical knowledge is very slight, if the operation is performed immediately after extraction. He never asks the patient, but proceeds as though it were part of the operation of extraction. He makes a longitudinal incison, and after removing such fragments of the gums as are partially detached, cuts away all prominent edges and irregular protrusions of bone.

Dr. Watling stated that in new cases he frequently removed considerable portions of bone and regarded it as possible in this manner to greatly improve the features.

Dr. Smith, during a discussion concerning the merits of Guillois' cement, stated that it is similar to all other bone fillings except that it hardens much more slowly and is perhaps a trifle more dense. He has seen os-artificial endure in one mouth four years with no more wearing away than we should look for in a tin filling. In another case it was entirely gone in two months. Thinks that the reported great success of Guillois' cement might have originated from some such a case of exceptional durability, and does not regard it as likely to fulfill the requirements of a permanent filling, as some gentlemen in the profession have seemed to hope.

Dr. Stone says that he has entirely revolutionized his mode of practice. Is using smooth points almost entirely for packing gold. Believes that he can make a more solid filling, and finish about the borders with less danger of breaking them, and has less dressing to do after the gold is all packed, for in fact the filling is finished all the time from the very start. He had had this question of smooth points introduced to him by Dr. Bannister, and believed it was destined to replace entirely the old serrated instruments. He believed that we used in general too many instruments. A few simple ones are all that are required for ordinary practice.

Dr. Smith in annealing gold regards passing it through the flame as the only method certain to remove all its impurities. Often a puff of white smoke or vapor, quite visible to the naked eye, will pass from the gold as it enters the point of greatest heat. The best operators have discarded the annealing tray, and are using the simple flame.

Dr. Barker stated that he had been in the habit of using smooth points for many years in connection with the common serrated points. Prefers them very much for filling retaining pits, remarking the fact that the pits need not be so deep. He makes his smooth points by filing away the serrations from common pluggers after drawing the temper, then carefully retempers and considers them fit for use.

Dr. E. S. Holmes was called to express his opinion on smooth points. He stated that he had nsed them during some months, and that he was highly pleased with the results. He regarded a cavity in a tooth as a wound to be healed. We cut away the diseased matter and dress this wound with appropriate medicaments; when the condition will warrant it we supply the lost tissue by carefully-placed new material; as the particles of gold a recarried to their destination the filling grows visibly under the instrument, and the wounded tooth is made whole again. Whether we use soft foil or adhesive foil, serrated points or smooth points, if we heal this wound the indications are filled. As the compositor was directed to follow the copy, even if it went out of the window, so we must follow the disease in a tooth through all its fissures and wherever it may lead—save all the tissues possible and take time to perfect results. I use Ney's gold from Nos. 4 to 60, also Johnson's rolled, No. 120, which works as easily as lead. With this I think we can restore lost tissue in a tooth as perfectly as with anything known. I pass my foil through the lamp. The property of cohesivesess in gold and modes of obtaining it I will say nothing about as they seem to have been very clearly set forth by Dr. Hawxhurst. The annealing of gold is simply to make it clean in order that cohesive attraction may re-establish itself under the instrument point.

Dr. Tremper read an excellent paper on dental education.

Dr. E. S. Holmes, during a discussion on the standard of dental education, stated that if ours was lower than the medical profession he did not wish to remain in it for a day longer.

Dr. Hawxhurst read a carefully prepared paper on the pulmonic absorption of noxious gases and odors. Some discussion of the subject followed, during which Dr. Hutchinson explained the advantage of breathing through the nose, and stated that the nose is nature's purifier of the air.

Jackson was chosen for the place of the next annual meeting.

Dr. Perry moved, and it was carried, that the Secretary be authorized to employ some eminent practitioner to give lectures and demonstrations to the association at its next annual meeting.

The officers elected for the ensuing year were as follows: President—Geo. H. Stone; Vice President—Geo. Mosher; Secretary and Corresponding Secretary—D. C. Hawxhurst; Treasurer—D. W. Smith; Censor—Dr. Watling.

Dr. E. S Holmes, the retiring President, delivered an able address, during which he showed the vivid contrast between dentistry as it existed twenty years ago and dentistry as represented by the brilliant achievements of modern times.

Then we used to hear about *plugging holes in teeth.* Now a cavity in the tooth is regarded as analagous to an ulcer in the soft parts, and the injury is regarded as a wound to be healed, and an amputation of the diseased part is effected, the cut surfaces of the cavity receive appropriate treament and the lost tissue is restored by art. The whole process involving a profound acquaintance with anatomy, physiology, chemistry, pathology, therapeutics, and even microscopy, such as would have been perfectly appalling to the poor itinerant toothplugger of the olden time.

The following standing committees were appointed: Anatomy—J. A. Watling, W. Owens, E. Hunter; Physiology—G. E. Corbin, F. W. Clawson, M. H. Knapp; Chemistry—D. C. Hawxhurst, J. C. Vaughan, M. C. Dorrance; Hygiene—G. P. Holmes, E. P. Cummings, J. W. Woolley; Pathology—D. W. Smith, G. H. Mosher, H. C. Phillips; Therapeutics—J. Douglass, J. W. Storm, E. G. Douglass; Surgery—B. Bannister, T. R. Perry, J. C. Parker; Education—G. R. Thomas, E. S. Holmes, R. S. Bancroft.

The next meeting of this association will take place at Jackston, November 12, 1872, holding in session three day.

D. C. HAWXHURST, Secretary.

### Eight Children at a Birth.

The *Boston Medical and Surgical Journal* states that, on the 21st of August, Mrs. Timothy Bradlee, of Trumbull County, Ohio, gave birth to eight children—three boys and five girls. They are all living, and are healthy, but quite small. Mr. Bradlee was married six years ago to Eunice Mowery, who weighed 273 pounds on the day of her marriage. She has given birth to two pairs of twins, and now eight more, making twelve children in six years. Mrs. Bradlee was a triplet, her mother and father being twins, and her grandmother the mother of five pairs of twins.

